# INCREASING VULNERABILITY AMONG OLDER AMERICANS ACT HOME-DELIVERED NUTRITION SERVICE CLIENTS

**DOI:** 10.1093/geroni/igz038.3432

**Published:** 2019-11-08

**Authors:** Kristen N Robinson, Heather L Menne

**Affiliations:** 1 Social & Scientific Systems, Inc., Silver Spring, Maryland, United States; 2 RTI International, Washington, District of Columbia, United States

## Abstract

Older Americans Act (OAA) programs are designed to help frail and vulnerable older adults remain in their homes through the provision of long-term services and supports. Administrative data from the Administration for Community Living (ACL) show that older adults receiving OAA services are three times more likely to live below the poverty level (33.0%) as compared with all older adults (9.2%). In addition, they are almost twice as likely to be living alone (45%) as compared with the general population (28%). Using data from the recently released 2018 National Survey of Older Americans Act Participants, we examine the largest program administered by ACL, the OAA Nutrition Program, to see if the economic vulnerability of home-delivered nutrition service clients has changed over the past 10 years. Results from this study show that recipients of home-delivered nutrition services are more in need of low-cost or free meals in 2018 than they were in 2008 due to a 24% increase in Medicaid eligibility, 41% increase in those who report not having enough money or food stamps to buy food, and 101% increase in those who report receiving food stamps. This increase in economic need may be due to a demographic shift in the marital status and living arrangements of older adults, specifically the 75-84 age group. The increase in the percentage of older adults who are divorced, live alone, and have low income has made the home-delivered nutrition services program even more important today than it was a decade ago.

